# Accuracy of wearables for determining the maximal oxygen uptake and lactate threshold: a qualitative systematic review

**DOI:** 10.3389/fspor.2025.1707991

**Published:** 2025-12-16

**Authors:** Lea Železnik Mežan

**Affiliations:** Faculty of Sports, University of Ljubljana, Ljubljana, Slovenia

**Keywords:** validity, reliability, physiology, activity trackers, smartwatches, athletes

## Abstract

**Introduction:**

The limitations of costly laboratory methods could be addressed by advances in wearable sensors and associated machine learning algorithms (13). Key advantages of wearable technologies (WT) include non-invasive and continuous monitoring. However, it is necessary to control and ensure their accuracy. The aim of this review was to analyze and systematically summarize recent studies on the validity of maximal oxygen uptake (V̇O_2max_) and lactate threshold (LT) as estimated by consumer wearables.

**Methods:**

The Preferred Reporting Items for Systematic Reviews and Meta-Analyses (PRISMA) guidelines were followed in preparing this review. The literature was systematically searched in the Web of Science, SPORTDiscus, and Medline databases. The risk of bias was assessed using the Quality Assessment of Diagnostic Accuracy Studies, version 2, tool.

**Results:**

Of the 252 records initially identified, 13 studies met the defined inclusion criteria. Most studies investigated Garmin smartwatches with compatible chest belt heart rate (HR) sensors and the Firstbeat Technologies algorithm. V̇O_2max_ was measured in all cases in the laboratory with a treadmill graded exercise test (GXT), while estimations were generated from submaximal outdoor runs. Studies with healthy untrained adults, recreational athletes and highly trained or professional athletes were equally represented. In seven studies wearable devices proved to be valid or acceptable for V̇O_2max_ estimation compared to the gold standard measurement. Three studies demonstrated validity of LT estimation.

**Discussion:**

Our review demonstrates valid estimations of V̇O_2max_ using WT in populations of healthy untrained adults, recreational athletes and team sports professional athletes. Usefulness in elite endurance sports is questionable and may depend on artificial intelligence (AI). Two or more submaximal runs as an index test could improve validity, but further examination is needed. An important finding is that valid estimations were calculated from submaximal tests. These make consumer wearables a user-friendly alternative to laboratory GXT, which has some limitations. To provide insight into stability of algorithms, there is a need for longitudinal studies that would monitor the accuracy of WT over months or even years. Ongoing research into the latest models of smartwatches and synthesis of data is critical to understanding their suitability and usefulness.

## 1 Introduction

Maximal oxygen uptake (V̇O_2max_) is the value of oxygen uptake per minute at which further increases in workload do not result in additional oxygen uptake (1). It is the maximum amount of oxygen that can be utilized by an organism during intense physical activity in one minute and represents human aerobic energy potential. Determining of V̇O_2max_ is currently the best method for assessing cardiorespiratory fitness in athletes and healthy untrained adults (2, 3). It enables health monitoring and performance evaluation. The lactate threshold (LT), also known as maximal lactate steady state, is the highest workload that can be maintained over time at which blood lactate production and clearance are balanced. It is defined as the workload at which blood lactate concentration does not increase by more than 1 mmol/L during the final 20 min of constant load exercise (4, 5).

Among the various metrics, V̇O_2max_ and LT are key performance indicators for athletes, especially in endurance disciplines (6, 7). The only direct way to measure V̇O_2max_ is open-circuit spirometry in a laboratory, which is the gold-standard method. In this procedure, lung ventilation and the exhaled fractions of oxygen and carbon dioxide are measured during a controlled exercise protocol, such as the treadmill ramp test (8, 9). The gold standard for determining LT is a test in which each participant performs several constant-intensity running bouts of approximately 30 min at intensities close to the estimated LT (5). During each run, lactate is measured every 5 min. However, these methods are laboratory-based, time-consuming, expensive and require specialized equipment and personnel (8, 10). They are also inconvenient and impractical due to the use of uncomfortable masks and invasive blood sampling (5, 10). In addition, determining V̇O_2max_ requires participants to exert maximal effort, which can disrupt athletes' training and recovery routines (11).

Thanks to technological advances, particularly in chip miniaturization (12), alternatives to various measurements in the form of modern wearable technologies (WT) are now available (8). The limitations of costly laboratory methods could be addressed by advances in wearable sensors and associated machine learning algorithms (13). There are also types of WT that can be used during running to track changes in muscle oxygenation in the calf or quadriceps muscles as a function of running speed (10). This method, called near-infrared spectroscopy (NIRS), is non-invasive, and its validity and reliability in detecting LT have been demonstrated (14, 15). However, its application remains costly and largely laboratory-dependent, requiring trained personnel for measurement and interpretation.

As technology has advanced, the functions of fitness trackers have expanded to include biomechanical and metabolic variables that were previously available only in laboratories for professional athletes and those able to pay for such tests (3). In recent years, engineers have aimed to develop new algorithms for predicting V̇O_2max_. One approach has been to exploit its relationship to heart rate (HR) and accelerometer features extracted during submaximal running (1). This method requires various body-worn sensors, such as HR monitors and accelerometers, positioned on different parts of the body (e.g., the tibia). A similar approach was used by Firstbeat Technologies Ltd (9), which integrated automatic V̇O_2max_ estimation into Garmin smartwatches. The most reliable data segments are used to estimate a person's V̇O_2max_ by utilizing either linear or nonlinear relationships between HR and speed data. These parameters are monitored continuously and automatically during each workout. Another method for predicting V̇O_2max_ involves extracting data based on resting conditions, such as heart rate variability (HRV) (16, 17).

Until recently, Garmin smartwatches estimated LT using a LT guided test (10), which was a type of graded running test. Currently, a range of smartwatches offer an “*Improved Lactate Threshold Measurement Feature”* that enables automatic detection of LT, eliminating the need for a guided test (5). Key inputs in the analysis of LT in Garmin smartwatches include the user's current V̇O_2max_ estimate, HR and pace data from recent runs, and race-time predictions (8).

Consumer-grade WT can track changes in V̇O_2max_ and LT over time (18). These features make WT a desirable alternative to standard laboratory testing methods. Furthermore, continuous monitoring of athletes' aerobic training adaptations provides potentially valuable information for athletes and coaches about the effectiveness of their training (3, 18). Through continuous monitoring, small changes in aerobic capacity can influence the training program (19). As these consumer wearables are not subject to regulation (20), there is no regulatory body to ensure their accuracy (8). Therefore, if researchers, athletes, coaches, health officials and healthcare professionals hope to continue using these devices, understanding of their accuracy and appropriate use is required. This underscores the importance of independent validation of WT by researchers to advance various scientific fields (20).

There are no universally accepted analytical techniques for measuring the accuracy or validity of WT (21). Carrier et al. (21) recommend that researchers seeking to validate a wearable device perform at least three analyses to assess validity: 1) a correlation test [such as Pearson, Spearman, Intraclass Correlation Coefficient (ICC), or Lin's Concordance Correlation Coefficient (CCC)], 2) Mean Absolute Percentage Error (MAPE), and 3) Bland–Altman plots with 95% limits of agreement. There are also no universally accepted thresholds for determining validity, particularly for less commonly used physiological and biomechanical variables, such as V̇O_2max_ and LT (3, 18). MAPE is often calculated because it provides a more conservative estimate of error, accounting for both overestimation and underestimation (22). However, as actual values approach zero, percentage errors can become extremely large even if forecasted values are close to actual values, which can distort the MAPE. Previous studies on WT have generally demonstrated the validity of V̇O_2max_ through a MAPE of less than 10% (and CCC ≥ 0.7) in field or outdoor settings (3, 18, 19, 21). Researchers suggest that thresholds for equivalence testing should be explored and refined if this method is to be used more widely.

Molina-Garcia et al. (16) reviewed the literature (up to January 14, 2021) on the estimation of V̇O_2max_ by consumer wearables. Their meta-analysis indicated that wearables using exercise-based algorithms provided more accurate V̇O_2max_ estimates than those based on resting conditions. Exercise-based estimation appeared optimal for measuring V̇O_2max_ at the population level, but estimation error at the individual level was large. The authors concluded that these methods still need improvement for sports or clinical purposes. They compared V̇O_2max_ estimates for 14 different wearables. However, frequent hardware and software updates in consumer devices may affect data quality. Given the rapid pace of innovation between 2021 and 2025, especially in optical sensing and adaptive calibration models, earlier conclusions may now be outdated. Therefore, it is important to regularly evaluate these devices for daily use (13) and to summarize the latest findings. On the other hand, since only a few smartwatches at that time (16) that could provide LT estimates, such collective comparisons for this parameter are still lacking (10).

We therefore pursued two objectives: 1) to review the most recent studies on the validity and reliability of V̇O_2max_ and LT estimated by wearable devices, and 2) to qualitatively summarize the findings to provide scientifically endorsed recommendations for future studies and potential users of these wearable devices.

## 2 Materials and methods

### 2.1 Literature search strategy

**Figure 1:**
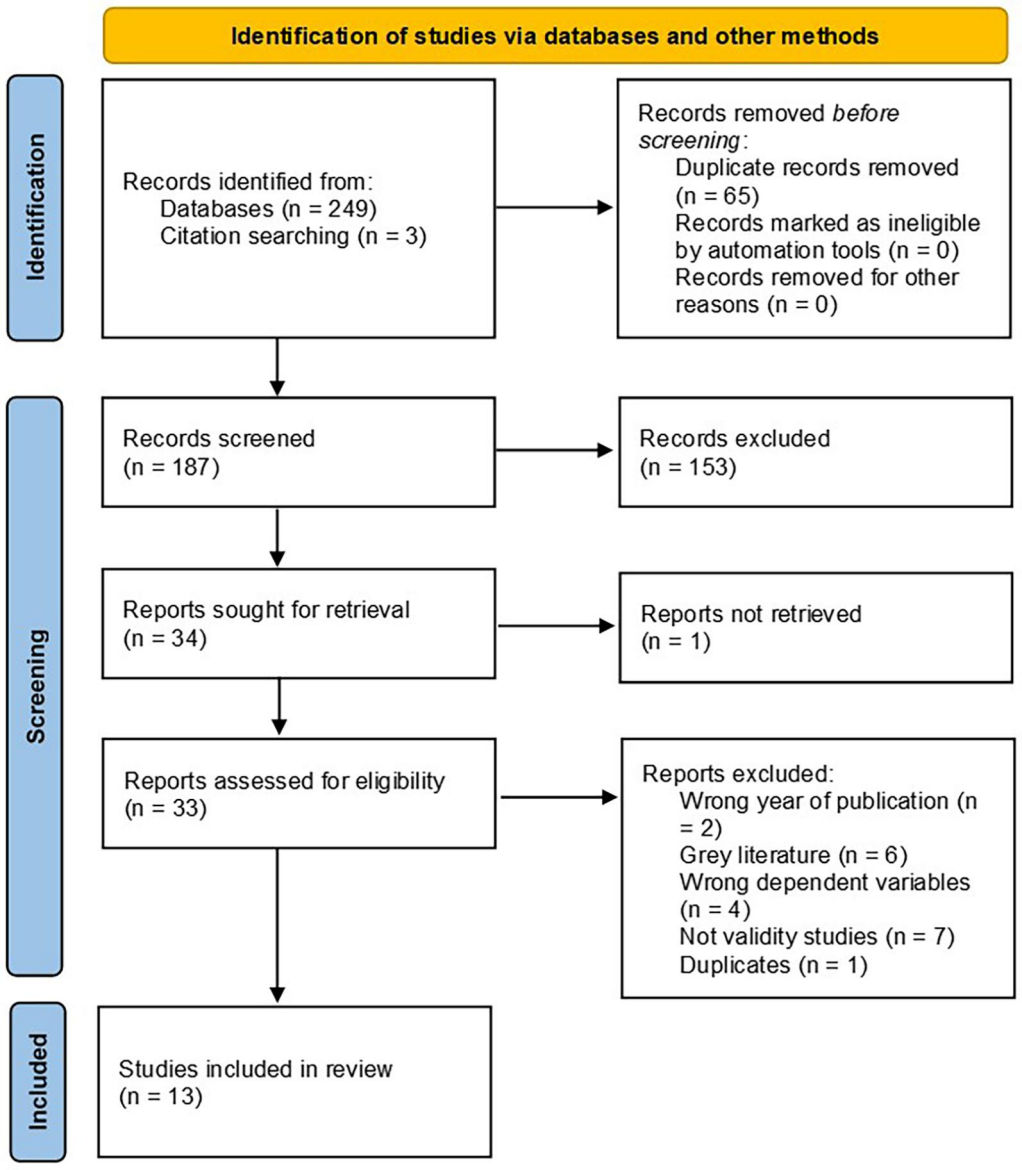
PRISMA flow diagram (23).

This review was conducted according to the recommendations of the Preferred Reporting Items for Systematic Reviews and Meta-Analyses (PRISMA) guidelines () and the Population-Intervention-Comparators-Outcomes (PICOS) design (23). The literature was systematically searched using the Web of Science, SPORTDiscus and Medline databases. Eligibility criteria were established following the PICOS approach, and the search strategy was defined as follows: 1) Population: healthy adults (≥16 years old); 2) Intervention: V̇O_2max_ and/or LT estimation by WT; 3) Comparison: V̇O_2max_ and/or LT gold-standard measurement; 4) Outcomes: validity and/or reliability of WT for V̇O_2max_ and/or LT estimation; 5) Study design: experimental and quasi-experimental designs. The search strategy is available for replication in Tables 1, 2. Additionally, a hand search was conducted using the same strategy to identify further studies (Figure 1).

**Table 1:** Search terms used in Web of science, SPORTDiscus and Medline databases.

Outcome	Device	Population/activity	Additional term
(“aerobic threshold*” OR “aerobic capacity” OR VO2 OR VO2max OR VO2peak OR “oxygen uptake” OR “O2 uptake” OR “oxygen consumption” OR “O2 consumption” OR “lactate threshold*” OR “anaerobic threshold*” OR “maximal lactate steady state” OR MLSS OR OBLA OR “race time prediction*”)	(wearable* OR “wearable activity tracker*” OR “wearable fitness tracker*” OR smartwatch* OR watch* OR garmin OR “wearable technology” OR “digital coach*”)	(sport* OR runner* OR running OR athlete* OR sportsmen)	*Only in Medline* (health* OR covid*)

The first three search terms were connected with Boolean operator “AND”, only in Medline the fourth term was added using Boolean operator “AND NOT”.

**Table 2:** Search strategy—filters used in Web of science, SPORTDiscus and Medline databases.

Filter	Database		
	Web of Science	SPORTDiscus	Medline
Searchable Fields	All Fields	All Fields	All Fields
Language	English	English	English
Date Range	01. 01. 2020-31. 12. 2025	01. 01. 2020–31. 12. 2025	01. 01. 2020–31. 12. 2025
Additional Filters	Category—Sport Sciences	Type of Source—Scientific Journals	/
N of studies included	78	96	75

### 2.2 Selection criteria

We included studies that met the following inclusion criteria: 1) V̇O_2max_ and/or LT were estimated using consumer wearable devices and measured using the reference standard; 2) criterion validity studies; 3) original articles published in peer-reviewed journals; 4) full text available in English; 5) articles reported sample and screening procedures (e.g., data collection, study design, instruments, and outcomes).

Studies were excluded from further analysis if they met any of the following exclusion criteria: 1) inappropriate dependent variables (measurement of V̇O_2max_ and/or LT was not the aim of the study); 2) V̇O_2max_ and/or LT were not estimated using consumer wearable devices; 3) not validity studies; 4) wrong populations (unhealthy adults, children, elderly); 5) dependent variables not measured during physical activity (e.g., television, video games, playing instruments); 6) not original articles or wrong publication types (reviews, conference abstracts or papers, surveys, opinion pieces, commentaries, books, periodicals, editorials, case studies, non-peer-reviewed texts, master's or doctoral theses).

### 2.3 Quality assessment

The risk of bias was assessed using the Quality Assessment of Diagnostic Accuracy Studies, version 2 (QUADAS-2) tool (24). This tool guides the assessment of risk of bias in diagnostic accuracy studies across four domains: patient selection, index test, reference standard, and flow and timing. We rated the risk of bias in each domain as high, potentially high, probably low, or low.

### 2.4 Data extraction

For each included article, the following information was extracted using Elicit (25) (Supplementary Material, Table S3): 1) author's name and year of publication; 2) aim of the study; 3) WT used (type, HR sensor); 4) sample characteristics (participants—athletes/non-athletes, number, mean age); 5) protocols used for V̇O_2max_ and/or LT estimation by WT and reference standard; 6) main findings (validity of WT for V̇O_2max_ and/or LT estimation—MAPE); 7) limitations; 8) suggestions for future studies.

## 3 Results

### 3.1 Search findings

Using the search strategy described in the methods section, 252 references were imported into Rayyan: AI-Powered Systematic Review Management Platform (26). A total of 116 duplicates were detected, and 65 references were deleted after resolution. Titles and abstracts of 187 references were screened. Some abstracts were so brief that we did not receive enough information, so we included them in the full-text screening. In the next phase, 33 full-text articles were imported into Rayyan (26) and screened for eligibility. 13 articles were retained for a final review. The detailed process with the exclusion criteria is shown in the PRISMA flow diagram in Figure 1.

### 3.2 Data extraction and synthesis

Information extracted from the 13 included articles, outlining the aim, methods, and key findings of each study, can be found in the Supplementary Material. Table 3 presents a synthesis of data on the type of WT, participant characteristics, protocols used for V̇O_2max_ and/or LT estimation and measurement, and data on V̇O_2max_ and/or LT validity.

**Table 3:** Synthesis of data.

Study characteristic	Methodology	Reference
Type of wearable	Garmin	(3, 8, 10, 13, 17–19)
	Other smart or sport watch	(17, 27)
	Other	(28–32)
HR sensor	Optical	(8, 13, 27)
	Chest belt	(3, 10, 17–19)
Participants	Healthy untrained adults	(10, 19, 27, 28)
	Recreational runners	(3, 13, 30)
	Highly trained and/or professional runners	(18, 29–31)
	Recreationally active people	(17, 32)
	Professional sportsmen (not runners)	(8)
Participants` mean age	Under 18	(8)
	18–30	(3, 10, 13, 17–19, 27–29)
	30–50	(30–32)
	50+	
Protocol—activity	Running	(3, 8, 10, 13, 17–19, 27, 29–32)
	Cycling	(10, 28)
Protocol—location	Laboratory	(3, 8, 13, 17–19, 27–32)
	Outdoor (field test)	(3, 8, 10, 17–19, 27, 29, 30)
Validity—V̇O_2max_	Valid (acceptable)	(3, 8, 17–19, 28, 29, 34)
	Overestimated	(3, 13, 17)
	Underestimated	(13, 27)
MAPE [%]—V̇O_2max_	<5	(8)
	5–10	(3, 13, 18, 19)
	>10	(27)
Validity—LT	Valid (acceptable)	(18, 29, 30)
	Overestimated	(32)
	Underestimated	(10)
MAPE [%]—LT	<10	(18)
	>10	(10)

#### 3.2.1 Participant characteristics

The total number of participants is 273 (181 men, 92 women) (see Supplementary Material). The mean number of participants per study is 21 (range: 12–44). Studies with healthy untrained adults, recreational athletes and highly trained or professional athletes are represented fairly equally (Table 3). Four studies involved non-athlete participants (10, 19, 27, 28). Four studies included recreational runners (3, 13, 29, 30), but used different inclusion criteria: Dearing et al. (30) required regular running training for the previous 3 months, a minimum frequency of 3 days per week, participation in competitive running events, and being free from injury and illness; Düking et al. (13) required running 2–3 times per week for 45 min at a self-perceived low intensity; Carrier et al. (3) and Apte et al. (29) did not provide information. Four articles also studied highly trained or professional runners (18, 29–31). Carrier et al. (18) included 21 athletes who met the following criteria: weekly running distance of 42.49 ± 22.96 km and V̇O_2max_ at the 95th percentile or above. The inclusion criteria for highly trained runners in the study by Ruiz-Alias et al. (31) were more than 3 years of regular running experience, more than 4 sessions per week, a 10 km time of 30–42 min, and being accustomed to running on treadmills. Apte et al. (29) divided their sample into recreational runners (15 participants) and highly trained runners (18 males) with a personal best below 90 min for a half-marathon. Dearing et al. (30) formed three groups according to competitive status: internationally competitive (*n* = 2), national class (*n* = 3), and competitive recreational (*n* = 15). The sample in this review consists mainly of participants aged 18–30 years (Table 3). Only one study tested male highly trained/national level soccer players with a mean age of less than 18 years (17.3 ± 1.3 years) (8). Three studies included older participants, with mean ages of: 39.5 ± 14.6 years (30), 30.7 ± 9.7 years (35), and 31.2 ± 14.9 years (male), 39.5 ± 15.4 years (female) (32).

#### 3.2.2 Technical features of wearable devices

The synthesis of data from the included articles showed that most studies on WT measuring V̇O_2max_ and/or LT were conducted with Garmin smartwatches (Table 3). Different models of Garmin smartwatches were analyzed: Garmin Forerunner 230 (17), 245 (8, 13), Garmin Fénix 3 (3), 6 (18, 19), and 7 (10). In addition, smartwatches from two other manufacturers were tested: Polar V800 (17) and Apple Watch Series 7 (27). Other types of WT are presented in the Supplementary Material.

As explained in the Introduction, algorithms for V̇O_2max_ and LT estimation include HR. In five of eight studies examining smartwatches, a chest belt sensor was used to measure HR (3, 10, 18–20), while three articles reported using an optical sensor.

#### 3.2.3 Protocols for V̇O_2max_ and LT estimate

We aimed to include in our literature review studies in which V̇O_2max_ and/or LT were estimated during some type of physical activity. Running was the predominant activity (Table 3). V̇O_2max_ was measured in all cases in the laboratory using a treadmill graded exercise test (GXT) with respiratory gas analysis. LT as a reference standard was measured with an LT field test (10, 18, 29) or GXT (18, 31, 32) in both cases measuring blood lactate. Furthermore, V̇O_2max_ and/or LT were mainly estimated from an outdoor submaximal run (3, 8, 13, 17–19, 27). Only Düking et al. (8, 13) performed more than one run—two (8) or three (13) outdoor runs at a constant pace without stopping (13). For the first two runs, participants were instructed to run for 30–60 min until exhaustion [subjective assessment or rate of perceived exertion (RPE) > 18 on the Borg scale (1, 20)]. For the third run, runners were instructed to run for 30 min until fully exerted (RPE = 20). In Düking et al. (8), out of the tested athletes and prior to any data analysis, 16 of 24 randomly selected athletes wore the smartwatch on a second occasion approximately 3–5 days after their first run.

The following studies share that the intervention test lasted between 10 and 15 min and was performed at an individually constant pace at least several minutes above 70% of the participant's maximal HR (HR_max_) (in some cases estimated, in some measured during the reference standard) (3, 8, 18, 19). In all these studies Garmin smartwatches were examined and these instructions came from the manufacturer. Caserman et al. (27) and Snyder et al. (17) determined only self-paced runs. While Polar was used in one study as a comparison to Garmin, Snyder et al. (17) also collected data on HRV. For the Garmin smartwatch to estimate LT, participants performed the LT test with the watch (10). This and the study by Apte et al. (29) were the only ones in the sample that conducted only field tests.

#### 3.2.4 Validity of wearable devices for V̇O_2max_ and LT estimate

The authors of seven studies (3, 8, 17–19, 28, 29) concluded that the analyzed wearable device was valid or acceptable for V̇O_2max_ estimation compared to the gold standard measurement (Table 3). MAPE was used in 54% of studies. Although comparing the devices solely based on this statistical measure provides an imperfect view of their validity, it does offer some comparative value. One study reported the validity of V̇O_2max_ as a MAPE of <5% (5). Four studies determined validity as a MAPE of 5%–10% (3, 18–20), and only one study significantly underestimated V̇O_2max_ compared to laboratory measurements, with a mean difference of −4.51 mL/kg/min and a MAPE of 15.78% (27).

Two studies from the sample (13, 27) found that smartwatches overestimated V̇O_2max_ (V̇O_2peak_) at low fitness levels and underestimated V̇O_2max_ (V̇O_2peak_) at higher levels. Although Düking et al. (13) investigated V̇O_2peak_, the study was included in the review because it provides interesting findings. The authors cautioned that the validity values should be interpreted carefully when applied to other populations, such as cardiac patients or elite athletes. They divided the sample into runners with low (V̇O_2peak_ ≤ 45 mL/kg/min), medium (V̇O_2peak_ 45–55 mL/kg/min) and high (V̇O_2peak_ ≥ 55 mL/kg/min) V̇O_2peak_ to assess whether validity differed between subgroups. The findings showed that when V̇O_2peak_ was between 45 mL/kg/min and 55 mL/kg/min, the smartwatch and the criterion had similar variability, but the criterion was more reliable in detecting changes outside this range.

Carrier et al. (18) investigated how well WT works in trained individuals with higher V̇O_2max_ values (above the 95th percentile for their age and gender) who achieve LT at higher running speeds, compared to untrained individuals. In this athletic sample, the Garmin Fenix 6 estimate of V̇O_2max_ compared to 1-minute average V̇O_2max_ values was accurate and valid (MAPE = 6.85%; CCC = 0.70). LT estimates were valid when comparing speed at LT (MAPE = 7.52%; CCC = 0.79) and speed at onset of blood lactate accumulation (OBLA). However, HR at LT was not considered valid, as the correlation analysis showed a CCC < 0.70. The Bland–Altman plots revealed a tendency for error as V̇O_2max_ increased, raising the question of what would happen in individuals with very high aerobic capacity (>60 mL/kg/min).

Nevertheless, five studies in the sample (10, 18, 29, 30, 32) investigated LT estimates from WT (Table 3). Three of these (18, 29, 30) demonstrated the validity of a device, which in two cases was not a smartwatch (29, 30). Apte et al. (29) examined whether using biomechanical parameters improves the prediction of maximal aerobic speed and speed at LT during a field test. Foot-worn inertial measurement units (IMUs) and a chest-worn global navigation satellite system (GNSS-IMU) were used. All performance variables, including speed at LT, were predicted with acceptable error using only biomechanical measures. Another study tested the Stryd power meter (30). The Stryd is a 9-gram foot pod that attaches to a running shoe and estimates power, pace, distance, vertical oscillation, cadence, leg spring stiffness and ground contact time (GCT). The authors aimed to investigate the effectiveness of the website-generated Stryd critical power (CP STRYD) as a meaningful parameter for runners. The results suggest that CP STRYD has high predictive power for running performance and is closely related to LT and OBLA. CP STRYD is equivalent to CP calculated with an established model, indicating its reliability. CP STRYD facilitates polarized training without the need for laboratory testing, with Stryd GCT being a useful field-based parameter.

Carrier et al. (18) identified biases in LT estimates in individuals with very high LT values or HR at LT significantly different from approximately 175 bpm. Another study in our review examined speed and HR at LT (10). The Fenix 7 underestimated pace at LT compared to the standardized blood lactate field test, with a significant difference (the pace calculated by the Fenix 7 at LT was 11.96% lower than the field test). The HR estimated by the Fenix 7 at LT was 1.71% lower (*p* > 0.05). Heiber et al. (10) indicated that the Fenix 7 LT estimates are sufficiently accurate for recreational athletes, but they assume that for professional athletes, it would not provide the differentiated data required for high-quality training management.

### 3.3 Quality assessment

The risk-of-bias assessment for each outcome is presented in Table 4. In summary, more than 75% of studies were at high or potentially high risk of bias in the Patient selection domain because they used a convenience sampling technique. However, five studies (13, 19, 28, 31, 32) were judged as potentially high or unclear in only one domain. One study (18) was judged as low or probably low risk in all domains related to bias. Almost all studies were at low or probably low risk of bias in the Index test and Reference standard domains. For the Reference standard, laboratory GXT was used in all cases. Although it was conducted before the Index test, the interpretation of results should be independent of the results obtained with the other method. As the Index test was generally reported to follow after an appropriate interval, only a small number of studies were identified as potentially high risk in the Flow and timing domain (8, 17, 27, 29, 30).

**Table 4:** Quality assessment.

Reference	Patient selection	Index test	Reference standard	Flow and timing
Düking et al. (8)	High	Probably low	Probably low	Potentially high
Dearing and Paton (30)	Unclear	Probably low	Probably low	Potentially high
Heiber et al. (10)	Potentially high	Low	Potentially high	Low
Hedge et al. (28)	Potentially high	Probably low	Probably low	Low
Düking et al. (13)	Potentially high	Probably low	Probably low	Probably low
Snyder et al. (17)	Potentially high	Probably low	Probably low	Potentially high
Caserman et al. (27)	High	Probably low	Probably low	Potentially high
Ruiz-Alias et al. (31)	Potentially high	Probably low	Probably low	Probably low
Parisi et al. (32)	Potentially high	Probably low	Probably low	Low
Carrier et al. (3)	Potentially high	Potentially high	Probably low	Probably low
Carrier et al. (18)	Low	Probably low	Probably low	Low
Carrier et al. (19)	Unclear	Probably low	Probably low	Low
Apte et al. (29)	Potentially high	Probably low	Probably low	Potentially high

## 4 Discussion

Our review suggests that using a Garmin smartwatch and performing a submaximal 10–15-minute outdoor run to estimate V̇O_2max_ and/or LT is a good option. This approach allows estimates to be made during athletes' normal training protocols without requiring a day off for testing, and the estimates also proved to be valid (3, 8, 17–19, 27). In this section, we discuss the findings regarding activity intensity, target audience, and number of runs.

### 4.1 Index test

Although direct measurement of V̇O_2max_ is considered the gold standard among sports physicians for determining an individual's fitness level, previous research has shown that V̇O_2max_ also has limitations (33, 34). Several V̇O_2max_ criteria are based on surpassing threshold values for the respiratory exchange ratio and HR during the exercise test, or threshold values for post-exercise blood lactate concentration, and are used as evidence that the subject has given maximum effort (34). However, the large between-subject variation in these variables means that many subjects will meet these criteria during submaximal efforts. This variation also means that some subjects may not meet a particular criterion even when maximum effort is given. This limitation may be even more pronounced for the HR criterion. V̇O_2max_ is limited by the variability of individual effort and is highly dependent on the extent to which participants are adequately motivated to reach their true maximum (33). Consequently, improvement in the methodology of existing criteria, or development of new criteria, is required (34). To be valid across experimental studies, new or improved criteria need to be independent of exercise modality, test protocol, and subject characteristics. One procedure that has shown potential for yielding valid V̇O_2max_ criteria is the verification phase, which consists of a supramaximal constant speed run to exhaustion performed after the incremental phase of a V̇O_2max_ test. This upgraded test might have the potential to measure V̇O_2max_ accurately, but it is not the most appropriate for different populations, such as older and/or unhealthy adults, highly trained athletes, and others.

Dugas et al. (35) compared V̇O_2max_ estimates for maximal and submaximal exercise tests in apparently healthy adults. The analysis showed that the indirect methods included overestimated the true V̇O_2max_. Nevertheless, the submaximal exercise tests provided a more accurate prediction of V̇O_2max_ compared to the maximal exercise tests when the American College of Sports Medicine running equation was used.

The Firstbeat method for V̇O_2max_ calculation is inherently submaximal. Garmin devices, using algorithms developed by Firstbeat Technologies (9), consistently reported MAPE values below 10%, demonstrating validity for V̇O_2max_ (3, 8, 17–19) and even LT (18) estimation. The Firstbeat method uses an age-based estimated HR_max_ in the calculation (9). Therefore, error in the HR_max_ estimate affects the accuracy of the V̇O_2max_ estimate. If HR_max_ is underestimated by 15beats/min, the error in the V̇O_2max_ result is approximately 9%. If the HR_max_ is overestimated by 15 beats/min, the error in the V̇O_2max_ estimate is 7%. If the person's actual HR_max_ is known, the error in the V̇O_2max_ estimate drops to 5%. According to these data, V̇O_2max_ estimates from WT could be even more accurate when measuring and entering an individual's true HR_max_. As noted above, HR_max_ is an even more delicate concept than V̇O_2max_. However, once measured as accurately as possible, it should not change as quickly over time as V̇O_2max_ might as a result of endurance training. Düking et al. (13) did not enter the participants' HR_max_ into the software, explaining that many recreational runners do not know their actual individual HR_max_. While the latest Garmin smartwatch models determine HR_max_ automatically, this may also limit comparability between studies (10). Comparability is also questionable in the context of HR sensor type. In three studies (8, 13, 27), of which two (13, 27) underestimated the validity of WT for V̇O_2max_ estimation, only a photoplethysmographic sensor was used. Measurement of HR with a chest belt sensor is more accurate than with an optical sensor, especially during high-intensity activities (36–38).

Another way to calculate V̇O_2max_ is without any exercise. In our review, there was only one study (due to our search strategy) that compared an exercise and a non-exercise test for estimating V̇O_2max_ (17). Snyder et al. (17) found that the Garmin Forerunner 230 was more accurate than the Polar V800 in predicting V̇O_2max_ in both sexes. Since Garmin uses submaximal runs along with other physiological parameters, it may appear that using a submaximal exercise test to predict V̇O_2max_ is more accurate than using HRV at rest in recreationally active individuals. Furthermore, the accuracy of the Polar estimate of V̇O_2max_ is questionable (17). Garmin devices have scientifically proven higher accuracy compared to other smartwatches (17, 27). However, the use of HRV to predict V̇O_2max_ may be more appropriate for sedentary (unhealthy) individuals. In addition, Rexhepi and Brestovci (39) developed a regression model to predict V̇O_2max_ without exercise using age, body weight, and resting HR as predictors. The measured V̇O_2max_ showed a significant correlation (0.69) with the predicted V̇O_2max_. The Paired Samples t-test showed no significant differences between the measured and predicted V̇O_2max_ (*p* = 0.78).

These findings align with the conclusions of a systematic review and meta-analysis by the INTERLIVE Consortium (16). Molina-Garcia et al. (16) found that wearables using exercise-based algorithms estimate V̇O_2max_ more accurately than those based on resting conditions. The meta-analysis suggests that wearables can generally be used at the population level to determine V̇O_2max_. Our review adds a summary of findings regarding another key performance indicator for athletes, namely LT.

Compared to V̇O_2max_ estimates, far fewer studies have investigated wearables as an alternative for LT assessment in recreational and professional athletes. Given the practical and non-invasive nature of estimation using wearable devices, and existing studies that have demonstrated the validity of WT for estimating LT (18, 29, 30), the estimated values of this physiological parameter could have significant potential for use in recreational and competitive endurance sports. Moreover, the gold-standard measurement for LT is actually more reliable than the method used for V̇O_2max_ measurement (33, 34). However, the LT test protocol is impractical for performance testing because it requires too many exercise bouts, even though it remains the gold standard for determining true LT (4). It is especially useful for validating indirect tests that aim to estimate LT. Three years ago, when Molina-Garcia et al. (16) published their systematic review and meta-analysis of V̇O_2max_ estimates from WT, only a few smartwatches could provide LT estimates. Now, there are many. For example, Garmin offers a range of smartwatches with an “*Improved Lactate Threshold Measurement Feature”* that enables automatic detection of LT, eliminating the need for a guided test (40). Therefore, LT estimation from WT should be thoroughly investigated in terms of different wearable devices, populations (age, training status), and other relevant factors.

### 4.2 Target audience

Most of studies in our review reported that the tested WT provide acceptable V̇O_2max_ and LT estimations. Therefore, we suggest using WT for healthy adults and recreational athletes. The use of WT (such as the Garmin Forerunner 245 or similar devices) for V̇O_2max_ and LT estimation is also sufficient for adapting training loads to individuals in professional team sports (8). In practice, professional team athletes participate in measurements only a few times a year, with the focus on sport-specific skills and abilities rather than on physiological parameters such as V̇O_2max_ or LT. To continuously track changes in cardiorespiratory fitness and adjust training plans appropriately, WT is more than adequate. However, the authors of four included studies (10, 13, 18, 27) cautioned that validity may differ when the sample consists of highly trained runners with higher V̇O_2max_ values or higher speed at LT. Further research is needed before WT can be used in elite endurance sports (10).

In recreational runners, minor differences between WT estimates and measurements from respiratory gas analysis and/or lactate testing may not cause significant problems (10). It is likely that estimated LT pace and HR still positively influence training, competition, and recovery compared to having no information on these performance metrics. The collected estimates provide a basis for classifying individual training intensity zones and designing training cycles. If deviations in LT pace and HR are consistent, possible training adjustments and performance changes over time can still be recognized. However, for competitive or professional endurance athletes, the same estimation error can have more significant consequences. Their optimal training load is often close to their maximum load. A significant underestimation of thresholds could result in inappropriate training stimuli being applied continuously, preventing athletes from achieving optimal performance gains within a given training period. Conversely, overestimation of LT pace or HR could lead to unplanned fatigue or overtraining symptoms. Therefore, V̇O_2max_ and LT estimates should always be interpreted alongside additional objective information from the WT that indicates the athletes' performance and health status (e.g., HRV), as well as in correlation with the RPE.

Balancing intense training with adequate recovery is essential for optimal athlete performance (41). Both insufficient and excessive training can negatively affect performance, but advancements in WT now enable more effective monitoring of training and readiness levels. Garmin developed Garmin Coach, an artificial intelligence (AI)–powered “coach” that creates easy-to-follow training plans, adapting daily based on the user's recovery and performance metrics when used with a compatible Garmin device. This approach optimizes training efficiency and success, resulting in above-average performance with minimal risk of burnout and injury (42). Nilsson et al. (41) combined data from Garmin smartwatches and a 9-item questionnaire (Readiness Advisor) application to evaluate readiness during a 39-day training period. Two groups were studied: one with daily adaptive modifications to training based on readiness level, and another with a static regimen where no changes were made regardless of readiness. Results showed that the adaptive group maintained a more consistent readiness score and demonstrated improved physiological responses and better performance metrics. In contrast, the static group showed no significant improvements. These findings suggest that adaptive training plans, driven by individualized data and AI analytics, can significantly enhance performance outcomes and physiological adaptations for runners. This means that even if a smartwatch slightly overestimates V̇O_2max_ and LT estimates, its use could still be safe and effective also for elite endurance athletes.

### 4.3 The influence of additional runs and other limitations

In the study by Düking et al. (8), the Garmin Forerunner 245 showed improved accuracy in V̇O_2max_ assessment after a second non-fatiguing run, with MAPE decreasing from 5.58% to 1.06%. Based on these results, it is recommended to perform at least two continuous submaximal runs with the smartwatch to enhance the validity of V̇O_2max_ assessment. This aligns with Apple's statement that a higher number of outdoor workouts increases the accuracy of V̇O_2max_ estimation (43). Garmin also recommends recording at least a few outdoor runs, calculating the cardiorespiratory parameters after each run, and combining them with previous values to obtain the most reliable data (44). Carrier et al. (3) identified it as a limitation in V̇O_2max_ validation that participants only ran once for 15 min. However, an earlier study by Düking et al. (13) did not show that MAPE decreases with an increasing number of runs. Düking et al. (8, 13) investigated different populations, which could be an important factor.

Since the algorithms used by WT are not disclosed in detail by the manufacturers, we can only speculate why MAPE could potentially decrease with an increasing number of runs (8). The type of runs could also impact validity and reliability, but currently there is no information on which kinds of outdoor runs (continuous or interval, intensity, duration) or other endurance activities could most improve the accuracy of V̇O_2max_ estimates from WT. It is also possible that the algorithms require a sufficient amount of individual data to calculate V̇O_2max_ accurately. Garmin claims that the watch can learn with increased use of the device (9). Additionally, at least a few runs are required before a user can train with the Garmin Coach. In some studies in our sample, the authors reported resetting the wearable device to factory settings before the index test. For the other studies, we have no information on whether they also did this and did not report it, or if they did not take this step. This could have an important influence on the validity of V̇O_2max_ and/or LT estimation.

There are additional issues that should be addressed, as they could limit the comparability of findings across different studies. Since companies use different algorithms to estimate cardiorespiratory parameters, and these algorithms are not available to researchers, the comparability of V̇O_2max_ and LT estimations by various WT is limited (17). The accuracy of these estimations depends heavily on the protocol of the index test (e.g., activity type, endurance method, number of repetitions, duration, intensity), which varies between different types of wearables and also between older and newer smartwatch models. The positioning of the HR sensor (optical or chest belt) and subject-related parameters (e.g., HR_max_; skin photosensitivity, melanin concentration, and pigmentation in the case of photoplethysmographic sensors) could also affect the findings (36).

The included studies had relatively small samples (8, 27, 29, 32), which raises questions about the statistical power and generalizability of the results (8). Although the WT market is evolving extremely fast, all the reviewed studies, despite being recent, did not examine the latest smartwatch models. Furthermore, the diversity of models limits the generalizability of the findings (8, 18).

There are also some limitations directly related to our review that may affect the interpretation of the findings. One is that the review was conducted by only one reviewer; two or more could have provided additional perspectives to the discussion. The research question could also have been limited to smartwatches only, as they predominate and represent a distinct field.

### 4.4 Future research directions

Future research will need to evaluate newer models of smartwatches, but this would be easier if manufacturers were required to disclose the details of their algorithms (8). This would also allow for comparisons between smartwatches from different manufacturers. There is a need for an easily accessible, independent database to succinctly characterize which devices may be used in specific scenarios, based on independent, peer-reviewed validation literature (21). This would be helpful for anyone seeking to use WT, from consumers using it for recreational or competitive purposes to academics and professionals conducting high-level research.

Future studies should investigate the validity of V̇O_2max_ and LT estimates in larger groups of individuals. In addition, the authors suggest using more heterogeneous data in the future (27), such as studying populations with different fitness levels (recreational and competitive sports), health statuses (clinical settings), older adults, children, and others (3, 8, 10, 32). Individuals with very high V̇O_2max_ (>60 mL/kg/min), or high speed at LT, or a HR at LT significantly different from approximately 175bpm should be included in future studies.

Further research is also needed to validate WT as a reliable method for predicting V̇O_2max_ and LT (17, 32). Future studies on WT should examine whether validity is affected by the number of index tests and by runs of different durations and intensities, as well as under varying weather and environmental conditions (13). It should also be investigated whether the device becomes more accurate over time as it receives more data to estimate V̇O_2max_ (3). There is a need for longitudinal studies that monitor the accuracy of WT over months or even years of use. This would provide insight into the stability of the algorithms.

To more accurately assess an athlete's performance score, it may be useful to integrate WT with other technologies that provide physiological and biomechanical measurements, such as NIRS and IMU. This multivariate performance assessment would enable more precise and personalized training planning and analysis. Currently, the application and interpretation of NIRS are costly and laboratory-dependent, requiring trained personnel. However, methodological solutions already exist that use AI for training adaptability, such as digital twins with additional information on an athlete's training readiness (41). AI-driven training with the help of WT holds significant promise and requires investigation across various sample groups and activities.

## 5 Conclusions

Estimating V̇O_2max_ and LT with WT appears promising. Studies in our systematic review have shown considerable validity for V̇O_2max_ and LT estimation using various models of Garmin smartwatches (5, 8, 17–19) and some other types of WT (28–30). However, since only one study has demonstrated the validity of LT estimation in a smartwatch (18), additional research would benefit users.

The findings suggest performing a submaximal 10–15-minute run to estimate V̇O_2max_ and/or LT accurately (3, 17, 18). There is a hypothesis that more outdoor runs with a smartwatch improve accuracy, but future research should address this. We recommend exercise-based V̇O_2max_ and LT estimation for healthy adults, recreational athletes, and team sports professional athletes. However, there is a lack of studies proving the accuracy of WT for use in elite endurance sports (10, 13, 18, 27). Although the validity of V̇O_2max_ and LT estimation from WT in highly trained runners is questionable, their performance outcomes and physiological adaptations could improve with AI and its capability for daily adaptive modifications of training according to the athlete's readiness level (41).

Validation of V̇O_2max_ and LT estimates by the latest models of consumer-grade wearable devices makes an important contribution to the scientific field. Proven validity is good news for users, while key advantages of WT are non-invasiveness and continuous monitoring. This represents an improvement in the availability of tracking physiological metrics, as it relies on submaximal exercise tests. Most wearables do not provide valid parameters in all scenarios and populations, but are marketed with bold claims due to a largely unregulated market (20). Therefore, ongoing research into the latest types of WT, independent validation, and regular synthesis of data are crucial to understanding their suitability and usefulness.
