# Supplementary material for: Accuracy of wearables for determining the maximal oxygen uptake and lactate threshold: a qualitative systematic review
Source: Front Sports Act Living. 2025 Dec 16;7:1707991. doi: 10.3389/fspor.2025.1707991 (PMC12748164; doi:10.3389/fspor.2025.1707991)
Supplement: Supplementary file 1 [file Datasheet1.pdf]

Supplementary Material. Extracted data (25).

Reference	Aim	Type of wearable	Sample	Protocols	Main findings
Düking et al. 2024 (8)	The aim of the study was to assess the validity of VO2max estimation provided by a consumer-grade smartwatch and the YoYo Intermittent Recovery Run 2 (YYIR2) in comparison to respiratory gas analysis.	Garmin Forerunner 245	Total number of participants: 24 - Gender: Male - Age: Mean age 17.3 ± 1.3 years - Height: Mean body height 178.1 ± 6.2 cm - Weight: Mean body mass 71.5 ± 8.6 kg - Training level: Highly trained/national level athletes (Tier 3) - Recruitment: From a youth soccer academy.	Treadmill test employing respiratory gas analysis - YYIR2 - Non-fatiguing warm-up run wearing a Garmin Forerunner 245 smartwatch.	The smartwatch showed improved accuracy in VO2max assessment after a second run, with a MAPE decreasing from 5.58% to 1.06%. - The YYIR2 had a MAPE of 4.2%, which was lower than the smartwatch's first run but higher than its second run. - Performing at least two runs with the smartwatch is recommended to enhance the validity of VO2max assessment.
Dearing and Paton 2022 (30)	Primary aim: To compare CP STRYD with a CP calculated using an established linear power-time model. - Secondary aims: To establish which lactate or gas exchange threshold is most equivalent to CP STRYD and to compare CP STRYD with standard laboratory performance tests as predictors of running performance.	Stryd foot pod	Number of participants: 20 - Gender distribution: Males (n = 16), Females (n = 4) - Competitive status: Internationally competitive (n = 2), National class (n = 3), Competitive recreational (n = 15) - Inclusion criteria: Regular running training for the previous 3 months, minimum frequency of 3 days per week, participating in competitive running events, free from injury and illness - Mean age: 39.5 years (SD = 14.6 years) - Mean weight: 71.4 kg (SD = 9.9 kg) - Mean body fat percentage: 14.3% (SD = 6.7%).	Laboratory GXT - Outdoor 1500 m and 5000 m time trials - Maximal Anaerobic Running Test (MART) - GXT.	CP STRYD is highly predictive of running performance and closely related to VT2 and OBLA. - CP STRYD is equivalent to CP calculated using an established model, indicating its reliability. - CP STRYD facilitates polarised training without the need for laboratory testing, with Stryd GCT being a useful field-based parameter.
Heiber et al. 2024 (10)	The aim of the study is to compare the estimates of individual running pace and HR at the LT provided by the Garmin Fenix 7 smartwatch with those obtained from a standardized capillary blood lactate field test using the modified Dmax method.	Garmin Fénix 7 ® smartwatch	Total participants: 26 - Female participants: 7 - Male participants: 19 - Age: 25.97 (± 6.26) years - BMI: 24.58 (± 2.8) kg/m² - Inclusion criteria: Adults, healthy.	Introduction to the smartwatch and design - Calibration phase - LT test with the watch - LT field test with blood lactate.	The Garmin Fenix 7 smartwatch underestimated the pace at LT by 11.96% compared to a standardized blood lactate field test. - The HR at LT estimated by the Fenix 7 was 1.71% lower than the HR calculated with the Dmax method, but this difference was not statistically significant. - The study suggests that the Fenix 7 provides sufficient accuracy for recreational athletes but may not be accurate enough for professional athletes.

Reference	Aim	Type of wearable	Sample	Protocols	Main findings
Hedge et al. 2023 (28)	The aim of the study is to test if a machine learning model can accurately predict dynamic VO2 across exercise intensities, including slower VO2 kinetics during heavy-intensity exercise compared to moderate-intensity exercise.	Tightly fitting sensor shirt (Hexoskin, Carr e Technologies, Montreal, Canada).	Total number: 15 - Gender: 8 men, 7 women - Age: 27 ± 5 years - Height: 1.70 ± 0.08 meters - Mass: 71 ± 11 kilograms - VO2peak: 42 ± 5mL/min/kg.	The test in this study refers to three different pseudorandom binary sequence (PRBS) exercise tests conducted on a cycle ergometer in a laboratory setting, with varying intensities: low-to-moderate, low-to-heavy, and ventilatory threshold-to-heavy.	The temporal convolutional network model accurately predicted VO2 kinetics during both moderate and heavy-intensity exercise with low bias and strong correlation with measured values. - The model predicted slower VO2 kinetics with increasing exercise intensity, which is a critical aspect of aerobic metabolism. - The study enables nonintrusive monitoring of cardiorespiratory dynamics using wearable sensors and machine learning techniques, making it accessible for broader applications.
Düking et al. 2022 (13)	Validate the VO2peak assessed by a smartwatch (Garmin Forerunner 245) against a criterion measure. - Discuss how VO2peak measurements can assist in evaluating and guiding training procedures for runners.	Garmin Forerunner 245 smartwatch	Total participants: 23 - Gender: 12 female, 11 male - Mean age: 23 ± 3 years - Mean body height: 173 ± 8cm - Mean body mass: 70.1 ± 11.2kg - Ethnicity: Caucasian origin - Training characteristics: 2-3 times per week for 45 min at a self-perceived low intensity.	The test is a treadmill ramp test used to determine VO2peak in runners. It involves a ramp protocol with increasing treadmill speed until volitional exhaustion, verified by specific physiological criteria, followed by a verification phase to confirm VO2peak values.	The study validated the VO2peak assessment by a Garmin Forerunner 245 smartwatch against a criterion measure, finding an overall MAPE of 5.7%. - The MAPE varied by VO2peak range: 7.1% for lower, 4.1% for medium, and -6.2% for higher VO2peak, indicating overestimation in lower and underestimation in higher ranges. - For VO2peak between 45 and 55 ml/min/kg, the smartwatch and criterion measure had similar variability, but the criterion measure was more reliable for detecting changes outside this range. Our experimental procedures differ from previously performed studies as we include reliability testing of the criterion measure as well.

Reference	Aim	Type of wearable	Sample	Protocols	Main findings
Snyder et al. 2021 (17)	The purpose of this study was to compare A max with that of predicted VO2max values obtained from the Polar V800 and the Garmin Forerunner 230 and to determine whether the accuracy of these estimates differs between sexes.	Polar V800 and Garmin Forerunner 230	Number of participants: 44 - Age range: 19-35 years - Gender distribution: 50% female - Health status: Free from metabolic, cardiovascular, and pulmonary diseases - Physical activity level: Low-and moderate-risk individuals capable of moderate to vigorous physical activity.	Polar V800: Polar Fitness Test (resting test measuring HR and HRV) - Garmin Forerunner 230: 10-minute self-paced outdoor run - Actual VO2max (A max): Maximal GXT on a treadmill.	The Polar V800 significantly overestimated VO2max in men but not in women. - The Garmin Forerunner 230 did not significantly overestimate or underestimate VO2max in either men or women. - The Garmin Forerunner 230 was more accurate at predicting VO2max than the Polar V800 for both sexes. Correlations between predicted and actual VO2max were significant for each device for each sex. Despite the significant associations between predicted and actual VO2max and lack of significant differences. The Bland-Altman plots show that the random error was quite large for both devices and for both sexes. Thus, we would recommend that users use these devices with caution and be aware of the error associated with the devices when using them in the field.
Caserman et al. 2024 (27)	The aim of the study is to compare the Apple Watch Series 7's performance against the gold standard in VO2max estimation and Apple's validation findings, and to assess the accuracy and reliability of VO2max estimation using the Apple Watch Series 7.	Apple Watch Series 7	Total participants: 19 - Female participants: 7 - Male participants: 12 - Age range: 18 to 63 years - Mean age: 28.42 years - Mean BMI for females: 23.09kg/m² - Mean BMI for males: 23.76kg/m².	Laboratory Test: GXT using a cycle ergometer and a metabolic gas analyzer. - Outdoor Test: Submaximal exercise test using the Apple Watch Series 7 during a 15-minute run at a self-selected pace.	The Apple Watch Series 7 significantly underestimated VO2max compared to laboratory measurements, with a mean difference of -4.51ml/kg/min. - The Bland-Altman analysis showed good overall agreement, but the intraclass correlation coefficient indicated poor reliability. - The Apple Watch overestimates VO2max in individuals with poor fitness levels and underestimates it in those with excellent fitness levels.
Ruiz-Alias et al. 2022 (31)	The aim of the study is to determine the location of CP concerning LT, respiratory compensation point (RCP), and VO2max using the 9/3 minutes Stryd CP test and a GXT.	Stryd running power meter (specifically, the Stryd Wind version)	15 participants - Age: 30.7 ± 9.7 years - Height: 1.75 ± 0.1m - Body mass: 70.6 ± 5.5kg - Lean mass: 48.5 ± 3.3% - Fat mass: 14.2 ± 5.6% - VO2max: 66.33 ± 7.20ml/kg/min - Inclusion criteria: > 3 years of regular running, > 4 sessions/week - 30-42 minutes in 10km - Accustomed to running on treadmills.	"9/3 minutes Stryd CP test" and the GXT used to determine CP, LT, RCP, and VO2max.	LT, RCP, and CP were located at 72.86%, 86.40%, and 88.71% of VO2max, respectively. - CP did not differ significantly from RCP in high-caliber athletes. - CP and RCP represent the same boundary in high-caliber athletes.

Reference	Aim	Type of wearable	Sample	Protocols	Main findings
Parisi et. al. 2021 (32)	The aim of the study was to test the validity of a wearable lactate threshold sensor (WLTS) against accepted methods of determining LT.	The wearable device is the BSXinsight XR2 running edition, a WLTS that uses LED lights to monitor changes in muscle oxygenation and is worn on the leg inside a compression sleeve.	Total participants: 12 - Male participants: 6 - Female participants: 6 - Age (Male): 31.2 ± 14.9 years - Age (Female): 39.5 ± 15.4 years - Height (Male): 172.7 ± 5.7cm - Height (Female): 164.2 ± 5.4cm - Weight (Male): 79.0 ± 8.1kg - Weight (Female): 64.8 ± 9.8kg.	Test: Individualized GXT on a treadmill - Measurements: HR, RPE, and blood lactate at rest, at the end of each stage, and post-exercise - Comparison: WLTS vs. OBLA and an increase in blood lactate of 1mmol/L.	The WLTS was found not to be a valid method for determining LT compared to traditional methods. - The WLTS overestimated the treadmill speed at which LT occurred compared to traditional methods. - The study had a limitation due to its small sample size.
Carrier et al. 2020 (3)	The purpose of this study was to determine the validity of the Garmin fēnix® 3 HR fitness tracker.	Garmin fenix 3	17 healthy, recreational runners (eight males, nine females, 24.8 ± 4.3 years, 73.6 ± 15.9kg, 174.3 ± 6.0cm, 19.9 ± 24.3km/week, 12.4 ± 15.1mi/week) were recruited and used for this study.	An indoor, graded treadmill VO2max test using a metabolic cart and an outdoor 15-min run on a track while wearing the Garmin fēnix 3.	It is reasonable to the authors of the current study that a MAPE of less than 10% and a correlation value greater than .7 properly demonstrate that the fēnix 3 HR can be used with enough confidence to provide accurate data to influence training decisions in recreational runners.
Carrier et al. 2023 (18)	The aim of the study is to determine the validity of wearable technology in estimating VO2max and LT in athletic populations.	Garmin fēnix 6 watch and HR monitor (Garmin HRM-Run)	Number of participants: 21 - Age: 24.24 ± 6.30 years - Gender: 11 male, 10 female - Height: 171.68 ± 8.01cm - Weight: 65.14 ± 9.41kg - BMI: 22.01 ± 1.91 - Fat mass: 17.04 ± 5.69% - Muscle mass: 39.25 ± 3.26% - Weekly running distance: 42.49 ± 22.96km - VO2max percentile: 95th percentile or above.	Laboratory Test: Treadmill-based GXT to determine LT and VO2max. - Outdoor Test: - First run: 10-15 minute run at above 70% of estimated HRmax to estimate VO2max. - Second run: GXT guided by the watch to determine LT.	The Garmin fēnix 6 watch showed acceptable agreement with laboratory measurements for VO2max and LT in athletic populations. - The watch was accurate for VO 2max when compared to 1-minute averaged values, with a MAPE of 6.85% and a CCC of 0.7. - The watch accurately estimated speed at LT and OBLA but not HR at LT.

Reference	Aim	Type of wearable	Sample	Protocols	Main findings
Carrier et al. 2025 (19)	The aim of the study is to evaluate the accuracy (validity) of VO2max estimates and blood oxygen saturation (BOS) measured via pulse oximetry using the Garmin fēnix 6 with a general population participant pool.	Garmin fēnix 6	VO2max testing: 19 participants (11 male, 8 female; age: 25.50 ± 5.26 years; height: 173.63 ± 9.08 cm; weight: 74.08 ± 14.16kg; BMI: 24.42 ± 3.21; fat mass: 22.14 ± 6.06%; muscle mass: 36.87 ± 4.58%; weekly running distance: 25.07 ± 23.65km) - Pulse oximetry testing: 22 participants (13 male, 9 female; age: 25.48 ± 6.02 years; height: 173.27 ± 7.70cm; weight: 68.88 ± 9.10kg; BMI: 22.91 ± 2.40kg/m 2 ; fat mass: 18.55 ± 7.05%; muscle mass: 38.73 ± 3.61%).	VO2max test: GXT and outdoor run compared to a metabolic system. - BOS test: Comparison of fēnix 6 readings under normoxic and hypoxic conditions to a medical-grade pulse oximeter.	The Garmin fēnix 6 provided accurate VO2max estimates, closely aligning with laboratory data (15s; 30s). - The device failed to accurately measure BOS under any condition. - The Garmin fēnix 6 is useful for estimating VO2max but not suitable for measuring BOS.
Apte et al. 2022 (29)	To extend the wearables-based approach to the Cooper test by assessing the relative contribution of running biomechanics to endurance performance. - To explore different methods for estimating the distance covered in the Cooper test using a wearable GNSS receiver. - To investigate whether the use of biomechanical parameters improves the prediction of sVO2max and sLT during the field test.	Foot-worn IMUs: Physilog 5 - Chest-worn GNSS-IMU: Fieldwiz	Highly trained runners: 18 males, age 27.7 ± 5.4 years, height 178.8 ± 4.8cm, weight 69.6 ± 10.1kg, personal best below 90mins for a half-marathon. - Recreational runners: 5 females, 10 males, age 31.5 ± 5.9 years, height 173.7 ± 9.9cm, weight 67.8 ± 14.7kg. - Age range: 18 to 50 years.	12-minute Cooper running test conducted on a 400m tartan track, where participants are instructed to cover the highest distance possible. This test is used to estimate VO2max and MAS, and to assess endurance performance and biomechanical metrics.	The study accurately estimated the distance covered during the Cooper test using a wearable GNSS receiver. - Biomechanical metrics alone were sufficient to predict performance variables like sVO2max, sLT, and average speed with acceptable error. - A biomechanical profile was developed to represent running technique and its evolution with fatigue, identifying different profiles for runners with highest and lowest endurance performance.

Notes. LT = lactate threshold, HR = heart rate, HRV = heart rate variability, $\dot{V}O_2$ = oxygen uptake ($\dot{V}O_{2max}$ - maximal, $\dot{V}O_{2peak}$ – peak), MAPE = mean absolute percentage error, GXT = graded exercise test, RPE = rate of perceived exertion, OBLA = onset of blood lactate accumulation
